# SpiroESTdb: a transcriptome database and online tool for sparganum expressed sequences tags

**DOI:** 10.1186/1756-0500-5-130

**Published:** 2012-03-08

**Authors:** Dae-Won Kim, Dong-Wook Kim, Won Gi Yoo, Seong-Hyeuk Nam, Myoung-Ro Lee, Hye-Won Yang, Junhyung Park, Kyooyeol Lee, Sanghyun Lee, Shin-Hyeong Cho, Won-Ja Lee, Hong-Seog Park, Jung-Won Ju

**Affiliations:** 1 Division of Malaria and Parasitic Diseases, Korea National Institute of Health, Osong 363-951, Republic of Korea; 2 Genome Resource Center, Korea Research Institute of Bioscience and Biotechnology (KRIBB), Daejeon 305-806, Republic of Korea; 3 University of Science and Technology (UST), Daejeon 303-333, Republic of Korea; 4 Department of Parasitology, Kyungpook National University School of Medicine, Daegu, 700-422, Republic of Korea; 5 Insilicogen, Inc. #909, Suwon, Gyeonggi-do 441-813, Republic of Korea †These authors contributed equally to this work

**Keywords:** Sparganum, Plerocercoid, *Spirometra erinacei*, Expressed sequence tags (ESTs), Database

## Abstract

**Background:**

Sparganum (plerocercoid of *Spirometra erinacei*) is a parasite that possesses the remarkable ability to survive by successfully modifying its physiology and morphology to suit various hosts and can be found in various tissues, even the nervous system. However, surprisingly little is known about the molecular function of genes that are expressed during the course of the parasite life cycle. To begin to decipher the molecular processes underlying gene function, we constructed a database of expressed sequence tags (ESTs) generated from sparganum.

**Findings:**

SpiroESTdb is a web-based information resource that is built upon the annotation and curation of 5,655 ESTs data. SpiroESTdb provides an integrated platform for expressed sequence data, expression dynamics, functional genes, genetic markers including single nucleotide polymorphisms and tandem repeats, gene ontology and KEGG pathway information. Moreover, SpiroESTdb supports easy access to gene pages, such as (i) curation and query forms, (ii) *in silico *expression profiling and (iii) BLAST search tools. Comprehensive descriptions of the sparganum content of all sequenced data are available, including summary reports. The contents of SpiroESTdb can be viewed and downloaded from the web (http://pathod.cdc.go.kr/spiroestdb).

**Conclusions:**

This integrative web-based database of sequence data, functional annotations and expression profiling data will serve as a useful tool to help understand and expand the characterization of parasitic infections. It can also be used to identify potential industrial drug targets and vaccine candidate genes.

## Findings

Sparganum is the plerocercoid larva of the genus *Spirometra erinacei *of pseudophyllidean tapeworms. Human sparganosis occurs occasionally through the ingestion of freshwater contaminated with proceroid-infected cyclops or through contact with plerocercoid-infected animal hosts, such as frogs and snakes. Sparganosis is reported in many countries but is most common in eastern Asia [1]. Ingested spargana have the ability to invade various organs, such as the eye, subcutaneous tissues, abdominal wall, brain, spinal cord, lung, breast, and others [2,3]. Human sparganosis can cause diverse symptoms, such as non-specific irritation, uncertain pain, tissue mass formation, or headache, or it can cause no symptoms that affect the internal organs [4]. Thus, sparganum has become an important parasite for human health, and research into this organism will greatly contribute to the identification and characterization of genes and pathways involved in parasite development and function.

For contemporary functional genomic studies, the significant efforts invested in the creation of integrative transcript databases of expressed sequence tags (ESTs) derived from full-length cDNA libraries have provided opportunities to direct gene discovery and functional analysis. However, in the absence of a fully sequenced genome, parasite transcriptome data have only been analyzed in a limited number of studies [5-8]. Therefore, the motivation for this project was to develop a database and tool that would provide parasite researchers with flexible access to parasite-specific information about sparganum that is relevant for studying the production of parasite proteins and identifying molecules involved in key biological pathways that might serve as targets for diagnostic markers and treatments for the control of sparganum.

### Database contents and construction

**Figure 1:**
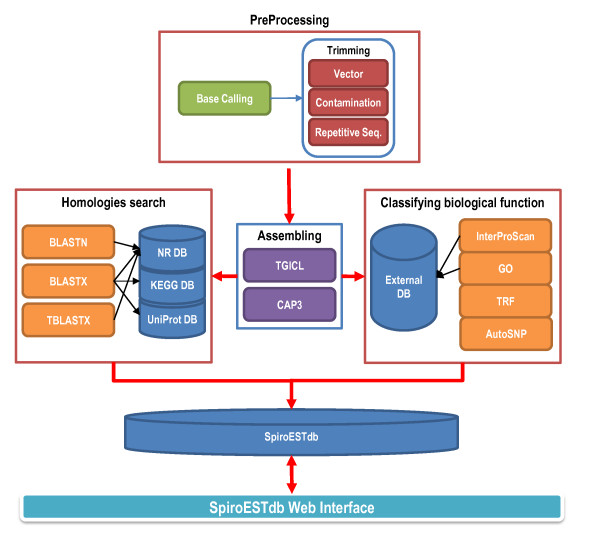
**Workflow schema of the pipeline**. The architecture of the pipeline includes four components: preprocessing for removing sequence contamination, transcript assembling for unigene clusters, homologies search and classifying biological functions. All data were deposited in SpiroESTdb. The User Interface (UI) supports end-user authentication, the profiling, selection and enactment of workflows.

To obtain a comprehensive view of sparganum gene activity, we constructed a non-normalized cDNA library derived from parasites isolated from wild snakes. In total, 5,760 randomly selected clones were picked and sequenced. After pre-processing, which included base calling and vector contamination and repetitive element trimming with Seqclean (http://www.tigr.org/tdb/tgi/software), we obtained 5,655 high-quality EST sequences greater than 100 bp in length using Phred score of 13 [9,10]. The sequences reported in this paper have been deposited in the NCBI database, under accession numbers HS514072-HS519705. We then assembled the 5,655 ESTs into 1,787 unigenes (910 contigs and 877 singletons) using the commercial software TGICL [11] and CAP3 [12] with the default parameters. To identify putative homology with known nucleotide and protein sequences, we performed separate BLASTN searches (Query Coverage and protein sequences, we performed separate BLASTN database, under -length cDNAaa, Identity range and protein sequences[13] for the 1,787 unigenes in the NCBI NR non-redundant nucleotide [14], KEGG [15] and UniProt [16] databases. To achieve better classification of the biological function of the unigenes, we also used InterProScan [17], Gene Ontology (GO) analysis [18], Tandem Repeats Finder (TRF) [19] and AutoSNP [20] (Figure 1). There were 1,262 unigenes (71%) shared homology with any other predicted or known molecules in public databases (Table 1).

**Table 1:** Summary of sparganum transcriptome

	Numbers
Total sequence reads	5,760

Total analyzed reads	5,655

Total assembled sequences (average size)	1,787 (725 bp)

Contigs	910 (787 bp)

Singlets	877 (661 bp)

Total annotated genes (non-redundant set)*	1,262

*Homologies search was performed by BLASTN, BLASTX, TBLASTX, Uniprot, KEGG and InterProScan

### SpiroESTdb architecture and web interface

SpiroESTdb is a relational database that uses an Oracle 11 g database management system and consists of a total of 22 tables. The database runs on a RedHat Enterprise Linux 5.5 platform with an Apache web server, and it was implemented with JSP (Java Server Pages), Java Servlet technology and the AJAX framework. The web interfaces were designed using HTML with some scripts written in JavaScript, and they use cascading style sheet (CSS) properties. The database is currently designed to work best with Microsoft Internet Explorer 8 (optimal resolution 1024 × 800). SpiroESTdb provides a user-friendly interface with seven main menus enabling access to (1) pre-processing reports, (2) clustering and assembling reports, (3) functional annotation reports, (4) curation for more accurate annotations, (5) expression profiling, (6) BLAST, and (7) a downloadable summary of all raw data and analyzed results.

## Utility and discussion

### General features of SpiroESTdb components and tools

#### Preprocessing information

**Figure 2:**
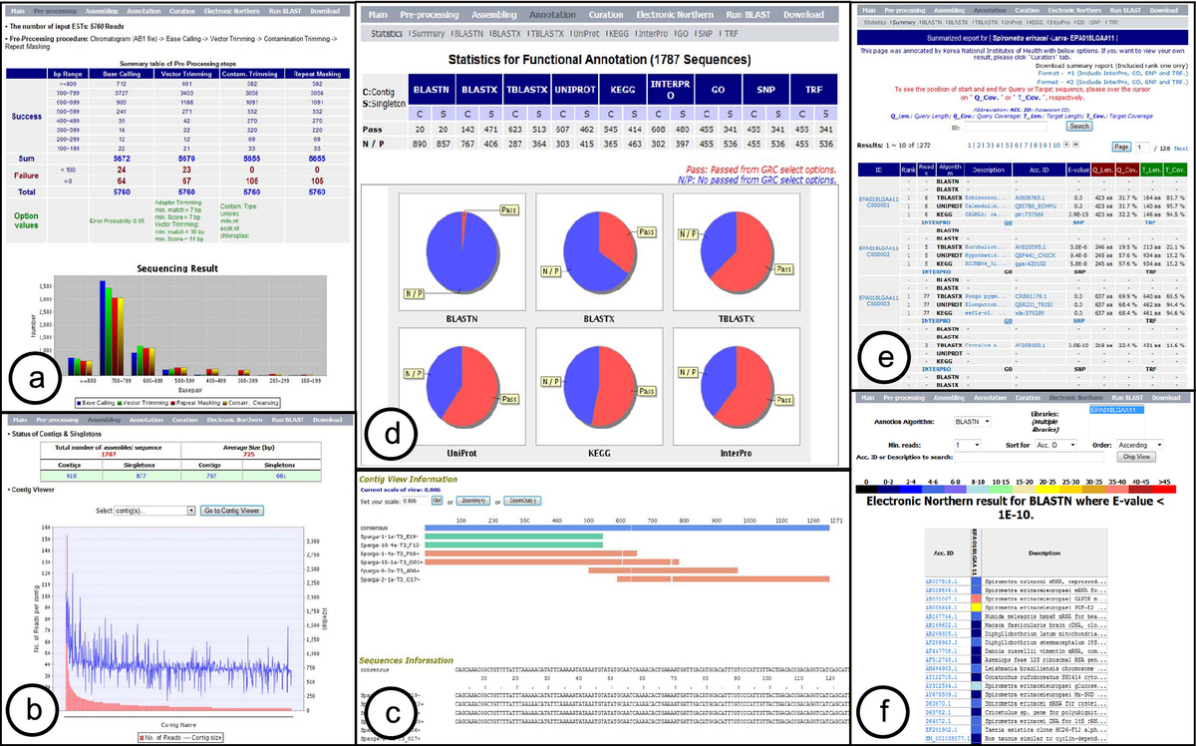
**Screenshots of the SpiroESTdb web application**. The panels show examples of results generated from general functions. (a) A pre-processing report showing cleansing sequence; (b) An assembly report for viewing contigs and reads; (c) Information of read distribution within a contig; (d) A statistics page describing an annotation result; (e) A summary page of various annotation results; (f) An example of secondary mining information showing *in silico *expression profiling information for all cDNA libraries.

To create the SpiroESTdb database, we employed a semi-automated annotation pipeline that uncovered genomic features in the raw EST sequences assigned for functional annotations based on the BLAST algorithms. First, the pre-processing information for generating high-quality consensus gene sequences is provided as a summary table in the pre-processing and assembling report (Figure 2a). The graphical display of these contigs with alignment sequence information and the trace viewer module allow for the evaluation of the raw sequencing data (Figure 2b and 2c).

#### Functional annotation

The online database of SpiroESTdb also provides comprehensive information on the ESTs through the "Annotation" section, which consists of 11 categories including Statistics, Annotation Report, BLASTN, BLASTX, TBLASTX, Uniprot, KEGG, InterPro, GO, SNP and TRF. In particular, in the case of the Statistics page, users can see all of the detailed functional annotation information describing how the unigenes were annotated and taxonomically distributed in the system (Figure 2d). Each category using the BLAST algorithm [13] allows users access to annotation information including unigene IDs and sequence information, annotated unique identifiers, sequence lengths, description, aligned start and end positions, matched similarity, and significant scores that were assigned by the prediction algorithms. SpiroESTdb allows users to view detailed alignment information by clicking on the description of each unigene (Figure 2e). InterProScan [17] is a predictive model for identifying protein domains, families and functional sites using diverse source databases. The InterPro category provides comprehensive information about the functional domains present in previously unidentified genes via a homology search. The potential assignment ID results are integrated into the GO category. As additional data, we also list single nucleotide polymorphisms in the SNP category and copy number variants in the TRF category.

#### Specific search function

Existing information can be retrieved for any unigene using full-text matching (against the read id, consensus id, gene name, gene accession number and functional description) in the "Annotation" page. With a gene or protein, users are able to blast a query sequence against raw read data, contigs and singletons, via the "Run BLAST" page, to find and visualize potential homologous matches.

### Personalized curation service and "Electronic Northern"

For some genes that have not previously been studied, the best hits identified using a homology search will provide ambiguous annotation descriptions such as "predicted protein" or "hypothetical protein". To increase the quality of the BLAST results and to offer biologically meaningful information, SpiroESTdb supports a user-friendly curation service that allows experts to manually edit functional annotations based on an experimental or heuristic approach. In addition, we programmed the "Electronic Northern" module to detect differential expression between different libraries and/or among unigenes within the same library by comparing accession IDs from BLAST results (Figure 2f).

### Practical examples using SpiroESTdb

**Figure 3:**
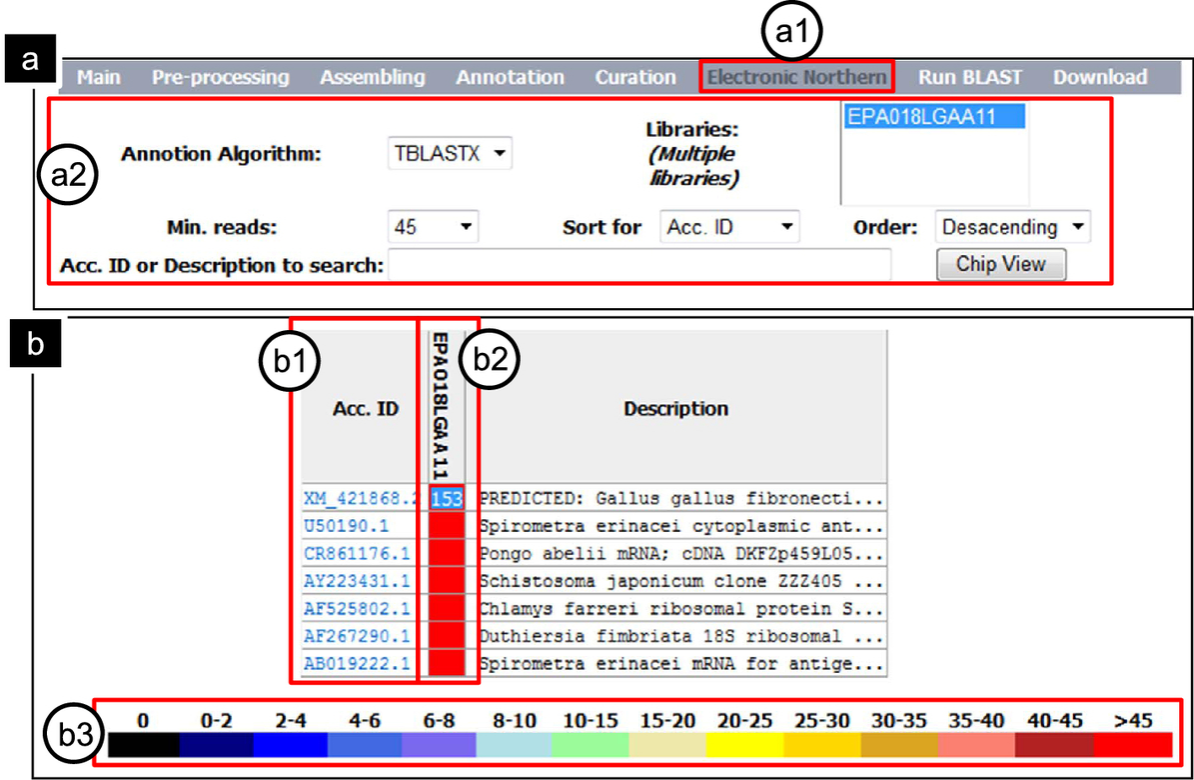
**A practical example using SpiroESTdb with "Electronic Northern"**. (a) The search interface. The steps involved in a "Electronic Northern" search are as follows: (a1) choose "Electronic Northern"; (a2) select search options for generating the appropriate output for your query, including "Annotation Algorithm", "Min.reads" and "Libraries". In the "Libraries" menu, the user can select a specific library; enter any additional information in the search field, if desired; and submit the query. (b) "Electronic Northern" results from a search for highly expressed genes. (b1) By selecting items under 'Acc.ID', detailed information can be obtained from the NCBI protein database. (b2) The number of reads can be displayed by double-clicking the center of the box in the corresponding color. (b3) The color spectrum indicates the expression level (the number of reads, n), ranging from low (black, n = 0) to high (red, n > 45).

SpiroESTdb includes a "Electronic Northern" module showing a profile of highly expressed genes that have been evaluated to determine the number of reads contained within one contig. The evaluation values are visualized as a spectrum of colors; black corresponds to the lowest value and red to the highest. Highly expressed genes can be considered novel genes with important biological functions for the parasite's survival and serve as drug targets for sparganosis treatment. For example, users can choose "Electronic Northern" on the sub-main menu and access several options; in the example shown, "TBLASTX" selection in the "Annotation Algorithm" field, the library selection in the "Libraries (Multiple libraries)" field and 30 or over in the "Min.reads" field (Figure 3a). The highly expressed gene with the most reads is a gene encoding *Spirometra erinacei *cytoplasmic antigen containing repeat epitope (U50190.1, 158 reads), followed by fibronectin (FN1, XM_421868.2, 153 reads), *Schistosoma japonicum *clone ZZZ405 mRNA sequence (AY223431.1, 90 reads), *Chlamys farreri *ribosomal protein S19 mRNA (82 reads) and *S. erinacei *mRNA for antigenic polypeptide (AB019222.1, 76 reads) (Figure 3b). In addition to these genes, a number of genes in the energy production pathway in the parasite, such as fructose-bisphosphate aldolase and glyceraldehyde-3-phosphate dehydrogenase, were highly expressed. Of the two antigens found to be highly expressed, one (U50190.1) must still be experimentally validated. FN is a ubiquitous and abundant glycoprotein that represents a combination of three independent domains, FN1, FN2 and FN3. Through interactions with different receptors, FN plays an important role in mediating cellular adhesion and migration processes, including embryonic development and wound healing [21]. Although the function of FN in the parasites is not clearly defined, FN is thought to have various functions that promote parasite survival in the host, such as providing a structural basis for cell adhesion, transducing signals for cell proliferation and apoptosis, and contributing to host defenses [22,23]. In other words, some information assigned by each annotation algorithm can be typed in "Acc. ID or Description to search" such as keyword, partial description and accession number.

## Conclusion

Further development of the database will involve updating the experimental records for verified sparganum (plerocercoid of *Spirometra erinacei*) genes with additional functional annotations, such as information about drug targets and vaccine candidates, maps of KEGG pathways and host-pathogen interaction information. Comparative genomes will also be integrated into SpiroESTdb to facilitate the production of large scale synteny maps and their associated genomic information. This database is available on the Web for all users upon registration and provides a large amount of information on potential industrial drug targets and vaccine candidates that can be used in virtual screening initiatives and molecular docking.

## Availability

SpiroESTdb is open access and freely available. The curation service requires free user registration because each user needs a unique session. All questions, comments and requests should be sent by email to todaewon@gmail.com.

Project name: SpiroESTdb

Project home page: http://pathod.cdc.go.kr/spiroestdb

Operating system: Linux

Programming languages: HTML, JSP, CSS3, JavaScript, AJAX, Oracle

Other requirements: None

License: None required

## Competing interests

The authors declare that they have no competing interests.

## Authors' contributions

DK designed and implemented the database and website and wrote the manuscript, and DK, WY, SN, JP and KL developed the web interfaces, assisted with the design of the database, performed database system administration, and integrated the bioinformatics tools in the application. HY, SC and ML helped with the preparation of the EST data sets (sample collection, cDNA library construction and sequencing). HP and JJ served as the principal investigators of the project. All authors contributed to the writing of the manuscript and have read and approved the final submitted version.

## Funding

This work was supported by grant 2009-0084206 from the Ministry of Education, Science and Technology, Korea and grant 2006-N54002-00 from the Korean National Institute of Health. The contribution of the staff at the Genome Research Center of KRIBB in Korea is gratefully acknowledged.
